# *Borrelia burgdorferi* adhere to blood vessels in the dura mater and are associated with increased meningeal T cells during murine disseminated borreliosis

**DOI:** 10.1371/journal.pone.0196893

**Published:** 2018-05-03

**Authors:** Ali Divan, Timothy Casselli, S. Anand Narayanan, Sanjib Mukherjee, David C. Zawieja, John A. Watt, Catherine A. Brissette, M. Karen Newell-Rogers

**Affiliations:** 1 Department of Biomedical Sciences, School of Medicine and Health Sciences, University of North Dakota, Grand Forks, North Dakota, United States of America; 2 Department of Medical Physiology, Texas A&M Health Science Center, College Station, Texas, United States of America; 3 Trauma, Health & Hazards Center, University of Colorado, Colorado Springs, Colorado, United States of America; University of Toledo College of Medicine and Life Sciences, UNITED STATES

## Abstract

*Borrelia burgdorferi*, the causative agent of Lyme disease, is a vector-borne bacterial infection that is transmitted through the bite of an infected tick. If not treated with antibiotics during the early stages of infection, disseminated infection can spread to the central nervous system (CNS). In non-human primates (NHPs) it has been demonstrated that the leptomeninges are among the tissues colonized by *B*. *burgdorferi* spirochetes. Although the NHP model parallels aspects of human borreliosis, a small rodent model would be ideal to study the trafficking of spirochetes and immune cells into the CNS. Here we show that during early and late disseminated infection, *B*. *burgdorferi* infects the meninges of intradermally infected mice, and is associated with concurrent increases in meningeal T cells. We found that the dura mater was consistently culture positive for spirochetes in transcardially perfused mice, independent of the strain of *B*. *burgdorferi* used. Within the dura mater, spirochetes were preferentially located in vascular regions, but were also present in perivascular, and extravascular regions, as late as 75 days post-infection. At the same end-point, we observed significant increases in the number of CD3+ T cells within the pia and dura mater, as compared to controls. Flow cytometric analysis of leukocytes isolated from the dura mater revealed that CD3+ cell populations were comprised of both CD4 and CD8 T cells. Overall, our data demonstrate that similarly to infection in peripheral tissues, spirochetes adhere to the dura mater during disseminated infection, and are associated with increases in the number of meningeal T cells. Collectively, our results demonstrate that there are aspects of *B*. *burgdorferi* meningeal infection that can be modelled in laboratory mice, suggesting that mice may be useful for elucidating mechanisms of meningeal pathogenesis by *B*. *burgdorferi*.

## Introduction

Lyme disease (LD) is a zoonotic bacterial infection caused by *Borrelia burgdorferi* that is transmitted to the host via the bite of an infected tick. With the exception of a bulls-eye rash, which does not present in all individuals, the acute symptoms of LD are non-specific, and flu-like. If antibiotic treatment is delayed, the infection can disseminate resulting in systemic infection and inflammation that can include regions of the central nervous system (CNS) [1, 2].

With respect to the neurological manifestations of LD, the NHP has been the most frequently used animal model. Using NHPs, multiple investigators have shown that tick infestation or infection with inoculum doses exceeding 10^7^ spirochetes, results in pleocytosis, lymphocytic meningitis, cranial neuritis and evidence of spirochetes in the CNS [3, 4]. Infection studies in NHPs also suggest that *B*. *burgdorferi* spirochetes have a tropism for the leptomeninges, and that pathogen burden increases with immunosuppression [5]. Meningeal thickening due to inflammation has also been reported in response to infection [6, 7].

Although the NHP has been demonstrated to be a suitable model of the neurological manifestations of LD, the main limitations of this model are the cost, training, and special ethical considerations associated with handling these animals [8]. While a handful of studies have provided evidence that spirochetes can occasionally be cultured out of CNS tissues in mice, CNS pathology has not been shown to occur as a consequence of intradermal or subcutaneous infection by *B*. *burgdorferi* [9–11]. In contrast, spirochetes belonging to the relapsing fever *Borrelia* species do seem to establish infection and cause pathology in the CNS [12, 13].

Recently, the dura mater, the most superficial layer of the meninges covering the brain, has been shown to contain lymphatic-like vessels that drain cerebrospinal fluid (CSF) and are responsible for the trafficking of leukocytes from the CNS to peripheral lymph nodes [14]. Given its role in leukocyte trafficking, the dura mater may play an important role in pathogen control and CNS homeostasis during infection. Notably, the dura mater also expresses decorin and multiple isoforms of collagen[15, 16]. Because *B*. *burgdorferi* is known to have tropisms for tissues that express decorin and collagen [17–19], we hypothesized that the dura mater is a tissue that *B*. *burgdorferi* colonizes during disseminated infection. In support of our hypothesis others have shown that the dura mater is colonized in mice infected by relapsing fever spirochetes [12], however there are currently no reports in the literature of such phenomenon occurring in mice infected by *B*. *burgdorferi*.

Given the importance of the dura mater in CNS immune cell trafficking, and the need for mouse models that replicate CNS manifestations of disseminated infection[8], the objective of our study was to determine whether any strains of *B*. *burgdorferi sensu stricto* colonized the dura mater during disseminated and late disseminated infection. We hypothesized that *B*. *burgdorferi* 297 colonized the dura mater during late stage dissemination and thereafter. Our results, presented below, demonstrate that *B*. *burgdorferi* is culturable when obtained during disseminated infection (45 days), and remains in the vasculature and other regions of the dura mater throughout late disseminated infection (75 days). Concurrent with the presence of spirochetes during late disseminated infection, we demonstrate significant increases in the number of T cells within the dura and pia mater of infected mice. Collectively, our results suggest that a mouse model may be appropriate for investigating certain aspects of *B*. *burgdorferi* meningeal infection and associated immune responses.

## Materials and methods

### Animals

Male C3H/HeN mice were purchased from Charles River or Envigo Laboratories All mice were housed in temperature and humidity controlled rooms, housed in 12h/12h light/dark cycles. All animals were 6 weeks old at time of initial needle inoculation. All animal work was reviewed and approved by institutional animal care and use committees at Texas A&M Health Science Center and University of North Dakota School of Medicine and Health Sciences.

### *B*. *burgdorferi* culture and infection

Low passage *B*. *burgdorferi* strain 297 [20] was purchased from ATCC. *B*. *burgdorferi* strain B31 clone MI-16 [21] was obtained as a gift from Brian Stevenson. To confirm the presence of plasmids that were required for infectivity, plasmid content for each strain of *B*. *burgdorferi* was analyzed by multiplex PCR with primers specific for regions unique to each plasmid, as previously described [22]. Spirochetes were cultured to mid-log phase in BSK-II medium at 37°C, 5% CO_2_, and quantified by dark field microscopy using a Petroff-Hausser chamber. Animals were placed under anesthesia using isoflurane, and infections were administered by injecting 100uL of inoculum intradermally into the dorsal thoracic midline[23]. Control animals were needle inoculated intradermally with 100uL of BSK-II medium.

### Tissue harvest and tissue culture

Prior to euthanasia, all mice were anesthetized by isoflurane. 50uL of blood was collected from the saphenous vein of each animal and cultured in 5 mL of BSK medium. After blood collection, control and infected mice were perfused transcardially with PBS and then 4% paraformaldehyde, using a peristaltic pump at a flow rate of 0.8mL/min for 6 minutes. Tissues were removed and aseptically transferred to 5mL of BSK-II medium containing 2.5 ug/mL amphotericin B and 50ug/mL rifampicin, and cultured in an incubator at 37°C, 5% CO_2_ for 42 days. Samples that did not have any spirochetes in 10 fields of view by day 42 of culture were considered negative.

### qPCR

Heart and brain tissues were isolated and immediately snap-frozen in liquid nitrogen prior to storage at -80°C. Tissues were ground under liquid nitrogen, and total DNA was extracted using DNeasy Blood and Tissue Kit (Qiagen 69506) following the manufacturer's instructions. DNA samples were then cleaned and concentrated using Genomic DNA Clean & Concentrator Kit (Zymo D4065). Quantitative PCR for the *B*. *burgdorferi flaB* and mouse *β-actin* genes was performed on each sample in triplicate, and absolute copy numbers interpolated using standard curves as previously described [24]. Data were log-transformed, and normalized to *flaB* copies per 10^7^ *β-actin* copies for each sample.

### Intravital tracers

Animals were anesthetized with isoflurane and injected retro-orbitally with 100uL of tracer dye. The tracer dye used was TRITC-conjugated 70 kilodalton lysine-fixable dextran (Invitrogen D1818), constituted to a concentration of 10mg/mL in PBS containing 2mM sodium azide. After retro-orbital injection, the tracer dye was allowed to circulate in the animal for 3 minutes, and each animal was either perfused transcardially as described above, or euthanized without perfusion. Dura samples were collected as described [25], and fixed in 4% paraformaldehyde overnight. On the following day, tissues were whole-mounted onto positively charged glass slides and cover-slipped in fluoromount-G with DAPI mounting medium (Southern Biotech 0100–20). Tissues were screened and imaged by epifluorescence using an Olympus BX51 microscope. Images were analyzed using FIJI-Image J software.

### Immunohistochemistry

Dura samples were collected from transcardially perfused mice by craniotomy, as described [25]. Each sample was post-fixed in 4% paraformaldehyde for 24h at 4°C. Samples were permeabilized in 0.1% Triton X-100, washed 3 times, and serum-blocked in 2.5% goat serum/PBS containing 1:100 dilution of Fc block (BD 553142). For *B*. *burgdorferi* staining, each sample was incubated in 1:100 dilution of rat anti-mouse unconjugated monoclonal anti-CD31 IgG (BD 550274), and 1:50 dilution biotinylated rabbit anti-*B*. *burgdorferi* polyclonal IgG (Invitrogen PA1-73007) at 4°C overnight. On the following day, the samples were washed, and stained with 1:100 dilution of Alexa 555 goat anti-rat polyclonal IgG (Invitrogen A-21434), and 1:200 dilution of Alexa 488 streptavidin (Invitrogen S11223) for 1 hour at room temperature, covered from light. Secondary antibody-only controls for *B*. *burgdorferi* indirect fluorescent assay were performed *in vitro* and no fluorescence was observed. Some of the dura samples were also stained for lymphatic vessels in a separate step, using 1:200 unconjugated rabbit anti-mouse polyclonal LYVE-1 IgG (abcam ab14917), followed by washing and secondary staining with 1:200 Alexa 633 goat-anti rabbit polyclonal IgG (Invitrogen A-21070). For CD3 staining, each sample was primary stained using 1:200 dilution of rabbit unconjugated polyclonal anti-CD3 IgG (abcam ab5690), or an equivalent concentration of rabbit unconjugated anti-mouse polyclonal IgG as an isotype control (abcam ab37415). Secondary staining was performed using 1:600 dilution of goat Alexa 488 polyclonal anti-rabbit IgG (abcam ab150081). CD31 staining was performed as described above. All brain samples were serially dehydrated in 10%/20%/30% sucrose, frozen in OCT (Tissue-Tek 4583), and cut on a cryostat in 50um sections. Representative sections were taken from each brain, and stained with antibody as described above. 50um sections of spleen from infected mice were processed in the same way as brain samples, as a positive control for CD3 staining. After antibody staining, all samples except those stained for LYVE-1, were incubated in PBS containing 1uM TOPRO-3 nuclear stain for 10 minutes, followed by 2 more washes. Each sample was onto a positively charged glass slide and cover-slipped in fluoromount-G with DAPI mounting medium (Southern Biotech 0100–20).

### Epifluorescence and confocal imaging

Spirochetes stained with Alexa 488 secondary antibody were identified by epifluorescence based on morphology and positive signal in the FITC channel using an Olympus BX-50 at 200x magnification. Cells that appeared to have spirochetal morphology but produced signal in any channel other than FITC were excluded from the analysis. Uninfected controls did not show any evidence of spirochetes in any regions of tissue. To confirm accurate morphology and to determine spatial distribution, spirochetes were imaged using a Ziess LSM 510 confocal microscope and the following settings: total frame averaging = 4, 488nm: Argon laser, power 5.0, bandpass filter 505-530nm, PMT 776, gain 1.0, offset 0.04, pinhole 1AU; 555nm: HeNe1 laser, power 20.0, bandpass filter 560-615nm, PMT 894, gain 1.0, offset 0.04, pinhole 1AU; 633nm: HeNe2 laser, power 30.0, longpass filter 650nm, PMT 825, gain 1.0, offset -0.02, pinhole 1AU. CD3+ cells stained with Alexa 488 secondary antibodies were identified at 200x magnification based on digital visualization of nucleated cells using Olympus Cell Sens software and the following settings determined by positive controls: FITC filter, ISO = 800, exposure time 250ms; DAPI filter, ISO = 800, exposure time 80ms; TRITC filter, ISO 800, exposure time 120ms. Of the CD3+ T cells that were identified at 200x magnification, additional images were taken at 400x and 600x magnification, and exposure times were adjusted to maximize signal/noise ratio. In intravital tracer experiments, all samples were imaged by epifluorescence at 200x magnification using the following parameters: TRITC filter, ISO = 800, exposure time 450ms, DAPI filter, ISO = 800, exposure time 120ms. All multichannel images were merged and analyzed using FIJI-Image J software.

### Leukocyte isolation and flow cytometry

The dura mater was removed from and leukocytes were freed from tissue by incubation for 30 minutes in 1mg/mL collagenase (Sigma C013-100mg) in HBSS supplemented with 5mM CaCl_2_. After incubation, the supernatant and remaining dura tissue was triturated with a syringe plunger through 100um strainers (Fisher 22-363-549). All cells were counted using a hemocytometer. Single cell suspensions were stained at 4°C for 20 minutes in a solution of 3% new born calf serum (NBCS) using the following antibody and stain dilutions: Aqua viability dye, 1:100 (Invitrogen L34966), PE CD45, 1:100 (ebioscience 12-0451-83), PacBlue CD3 1:100 (Biolegend 100214), FITC CD4 1:100 (BD 553651), or APC CD8 1:100 (Biolegend 100712). Samples were washed in 3% NBCS, and run on BD FACS CANTO II flow cytometer. Fluorescence compensations were performed using BD CompBeads (BD 552845), and aqua compensation was performed using ArC Amine reactive compensation bead kit (Life technologies A10346). All gating was done based on fluorescence minus one (FMO) controls. Flow cytometric data was analyzed using FlowJo software.

### Statistics

Cells in dura mater whole-mounts were quantified by manually counting all cells of interest in every 20x field of view. Cells in brain and pia mater were quantitated by taking 20 representative samples from 240 50um sections and manually counting the number of cells of interest in each section. Total cell numbers in brain and pia sections were computed by adjusting the number of cells counted in the representative sections for the total number of sections in each brain. All data were analyzed and graphed using GraphPad Prism 7.0 software. Power analysis was performed prior to performing the experiments using GPower3 software and the following parameters: a priori analysis, difference between two independent means, β = 0.20, α = 0.05, and an effect size of 2.2 (based on preliminary data). Unpaired t-tests were used to determine differences between control and infected groups, using α = 0.05.

## Results

### Spirochetes colonize the dura mater

**Fig 1:**
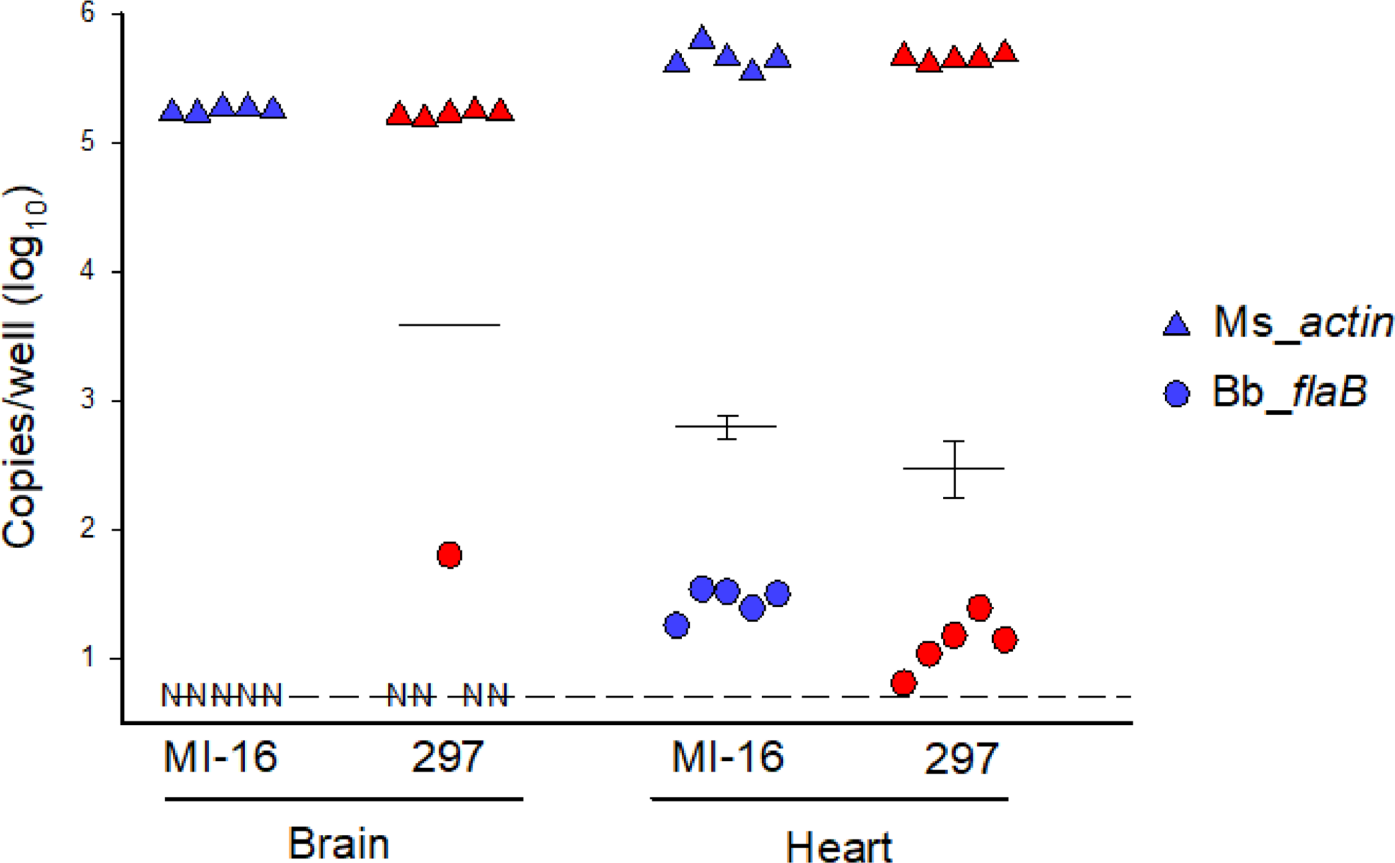
*B*. *burgdorferi* infects brain infrequently. Quantitative PCR of DNA isolated from brain and heart tissue isolated from mice infected with B31 MI-16 (blue symbols) or 297 (red symbols) as indicated. Copies/well for each biological replicate are shown for the *B*. *burgdorferi flaB* target sequence (circles), as well as mouse *β-actin* reference target (triangles). Horizontal lines indicate mean normalized *flaB*/10^7^ *β-actin* ± SD for each group. Dashed line indicates lower limit of detection. Samples with no detectable *flaB* signal are denoted by the symbol N.

In mouse model systems, spirochetemia is first observed within days of infection, and by 2 weeks after infection is detectable in all infected mice [26]. During the period of spirochetemia the infection disseminates to multiple organs and tissues, reaching its peak burden approximately 3–4 weeks after infection, depending on the tissue [26]. Initially, we infected mice intradermally with 10^6^ *B*. *burgdorferi*, strain 297, a clinical isolate derived from the cerebrospinal fluid of a patient [20]. To determine whether the infection had disseminated, and to test for the presence of spirochetes in the blood, we cultured blood samples from all mice 45 days post-infection (dpi), followed by transcardial perfusions with PBS to remove the remaining blood from circulation. The rationale for performing transcardial perfusions was to utilize shear forces to remove any non-adherent spirochetes from blood circulation, thus providing an accurate assessment of vascular adhesion and tissue colonization. After perfusion, fractions of ears, hearts, tibiotarsal joints, dura mater, and brains of all mice were cultured in BSK medium for a duration of 42 days. We observed that the ears, hearts, tibiotarsal joints, and dura mater were culture-positive in all mice (n = 5), demonstrating that the infection had disseminated (Table 1). 1/5 brain samples were positive for spirochetes, and all blood cultures were negative. We repeated the experiment using the same inoculum dose (10^6^ spirochetes) of a tick-isolated strain of *B*. *burgdorferi* called B31, and again using a lower inoculum dose (10^4^ spirochetes) of *B*. *burgdorferi* strain 297 (Table 1). In mice infected with 10^4^ of strain 297, 3/5 dura samples were culture positive, however these differences were not statistically significant from the data obtained using 10^6^ spirochetes of the same strain. The 10^6^ dose of strain B31 paralleled the data that we obtained using 10^6^ of strain 297, suggesting that spirochete colonization of the dura mater was not unique to strain 297. Consistent with our culture data, all heart samples were positive for *B*. *burgdorferi* genomic DNA by qPCR, whereas only one brain tissue from a mouse infected by strain 297 resulted in detectable signal using *B*. *burgdorferi*-specific primers (Fig 1). Thus, it seems that in mice, the brain is an infrequent target of *B*. *burgdorferi* infection. In contrast, the dura mater is a tissue that is consistently colonized by *B*. *burgdorferi* during disseminated infection, independent of the strains tested.

**Table 1:** Dura mater is colonized by spirochetes during disseminated borreliosis.

Borrelia Strain	297	297	B31
Inoculum dose	10^6^	10^4^	10^6^
Tissue	Number culture positive / total number of samples		
Ear	5/5	5/5	5/5
Heart	5/5	5/5	5/5
Tibiotarsal joint	5/5	5/5	5/5
Dura	5/5	3/5	5/5
Brain	1/5	0/5	2/5
Total	23/29	18/30	24/30
No. Infected/total mice	5/5	5/5	5/5

All mice were infected for 40 days

Blood cultures were negative in all mice

### Dura mater spirochetes preferentially adhere to blood vessels

**Fig 2:**
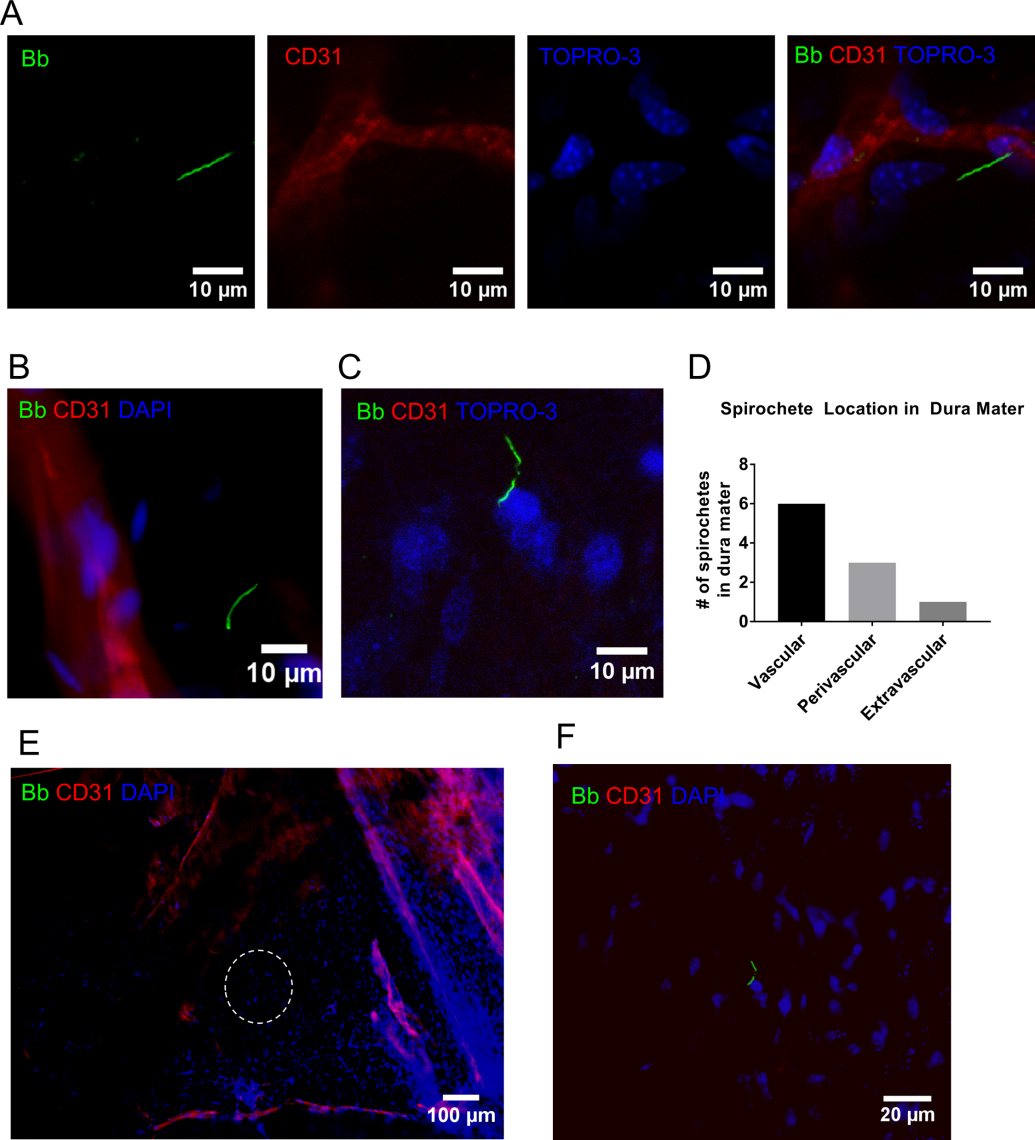
*B*. *burgdorferi* in dura mater during late disseminated infection. (A-C) Representative images of *B*. *burgdorferi* (Bb), blood vessels (CD31), and nucleated cells (DAPI or TOPRO-3) in regions of the dura mater, 75 dpi. (A) Confocal images described from left to right. *B*. *burgdorferi* (Bb) shown in 488 channel; CD31+ blood vessels in 555 channel; TOPRO-3+ nucleated cells in 633 channel; merged image showing *B*. *burgdorferi* in association with a blood vessel. (B) Epifluorescence image of *B*. *burgdorferi* in perivascular region near a blood vessel. (C) Confocal image of *B*. *burgdorferi* in extravascular region of dura mater. (D) Cumulative sum of spirochete locations in 3 dura mater samples; differences in (D) were not statistically significant. (E) 100x magnification epifluorescence image showing blood vessels (CD31) in the dura mater, but not within the region where the spirochete shown in (C) was detected (dashed circle). (F) 400x magnification of circled region in (E) showing spirochete (Bb).

To determine the anatomical location that spirochetes colonized the dura mater, we performed fluorescent IHC on samples that were isolated from transcardially perfused mice 75 dpi. Given that the dura mater expresses decorin, and multiple isoforms of collagen, we hypothesized that the majority of the spirochetes would be found in the extravascular spaces where these proteins are in relatively high abundance[15, 16]. We defined vascular spirochetes as touching or within vessel boundaries, perivascular as within one vessel diameter of the nearest vessel but not vascular, and extravascular as neither vascular nor perivascular. The majority of the spirochetes that we identified were found adhering to vascular and perivascular regions (Fig 2A and 2B), however we did observe spirochetes in extravascular regions (Fig 2C–2F). At this time point, we did not observe any spirochetes adhering to the lymphatic-like vessels in the dura mater that were stained with LYVE-1 antibody (S1 Fig). To exclude the possibility that transcardial perfusions were not effective in the dura mater, we injected mice intravenously with a 70 kilodalton TRITC-conjugated dextran, and compared fluorescent staining of vessels with and without transcardial perfusions. Our results demonstrate that the methodology that we used for perfusions was effective for removing non-adherent macromolecules in circulation (S2A and S2B Fig). Given that brain samples were occasionally culture-positive at 45 dpi (Table 1), we sought to determine if any spirochetes could be detected by IHC in representative brain sections at 75 dpi. Entire brains were cut into 50um sections, and 20 representative sections were stained to detect spirochetes. Of the samples that we screened, none of them showed any evidence of spirochete colonization of the brain, brain vasculature, or the attached pia mater (n = 3) (not shown). These results demonstrate that during late disseminated infection, spirochetes colonize multiple regions of the dura mater, and are predominantly found adhering to blood vessels.

### Increased T cells in meninges during infection

**Fig 3:**
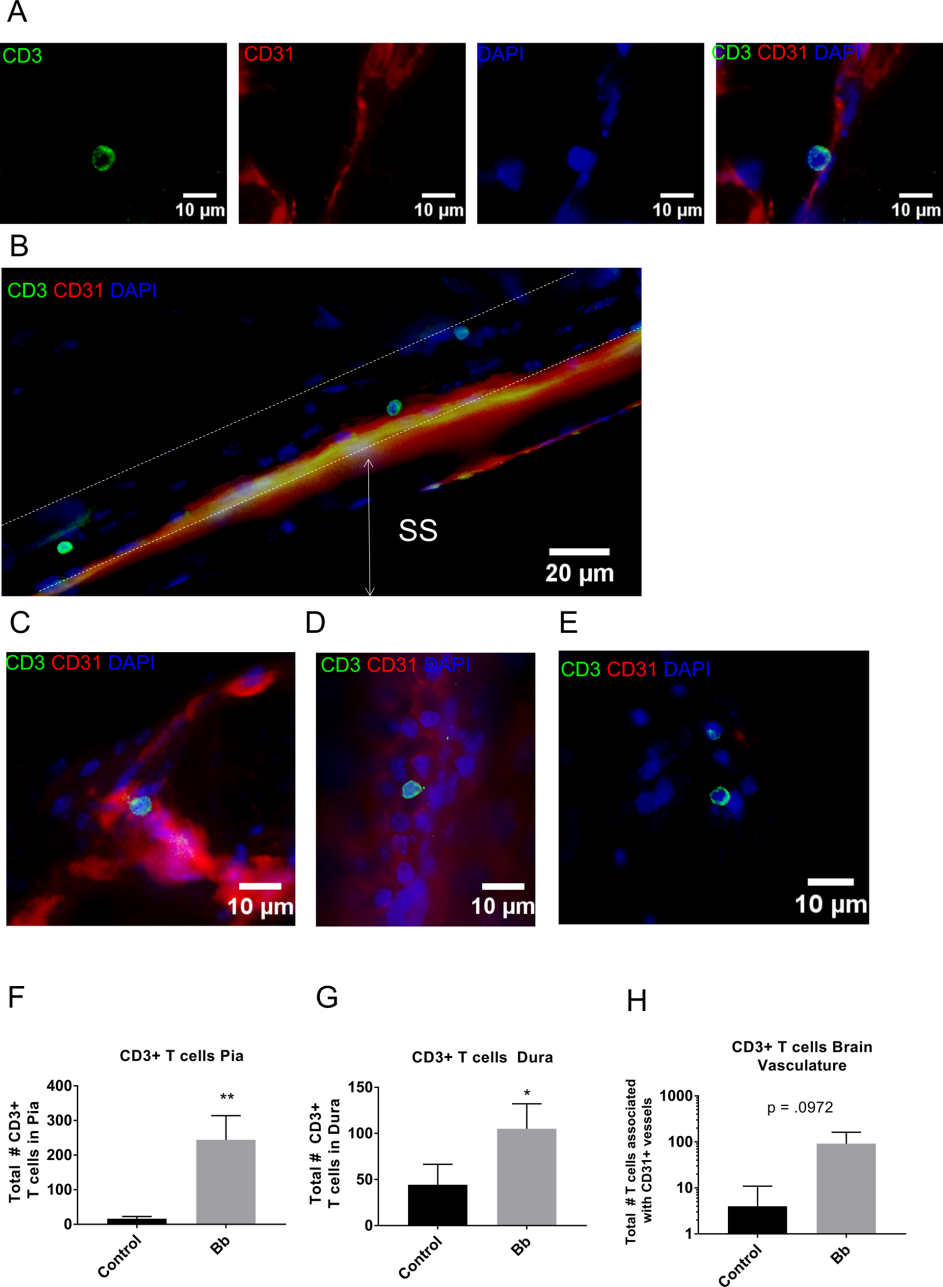
T cells in central nervous system during late disseminated infection. (A-E) Representative epifluorescence images of T cells (CD3), blood vessels (CD31), and nucleated cells (DAPI) in the brain, dura mater, and pia mater. (A) Epifluorescence images described from left to right. CD3 shown in FITC channel; CD31+ blood vessels shown in TRITC channel; nucleated cells shown in DAPI channel; merged image showing CD3+ cell associated with pia mater within the commissure of the isocortex. (B) T cells within the lymphatic-like vascular region of the sagittal sinus in the dura mater. (C) T cell associated with a blood vessel in the vasculature of the brain choroid plexus. (D) T cell associated with blood vessel in the dura mater. (E) T cell in extravascular region of the dura mater. (F) Total number of T cells observed associated with the pia mater of control and *B*. *burgdorferi*-infected (Bb) mice; n = 3, p = 0.0050. (G) Total number of T cells observed in the dura mater of control and *B*. *burgdorferi*-infected mice; n = 3, p = 0.0403. (H) Total number of T cells in brain vasculature of control, and *B*. *burgdorferi-*infected (Bb) mice; n = 3, p = 0.0972; Statistics computed using t-test, α = 0.05; *p≤ 0.05, **p≤ 0.005.

**Fig 4:**
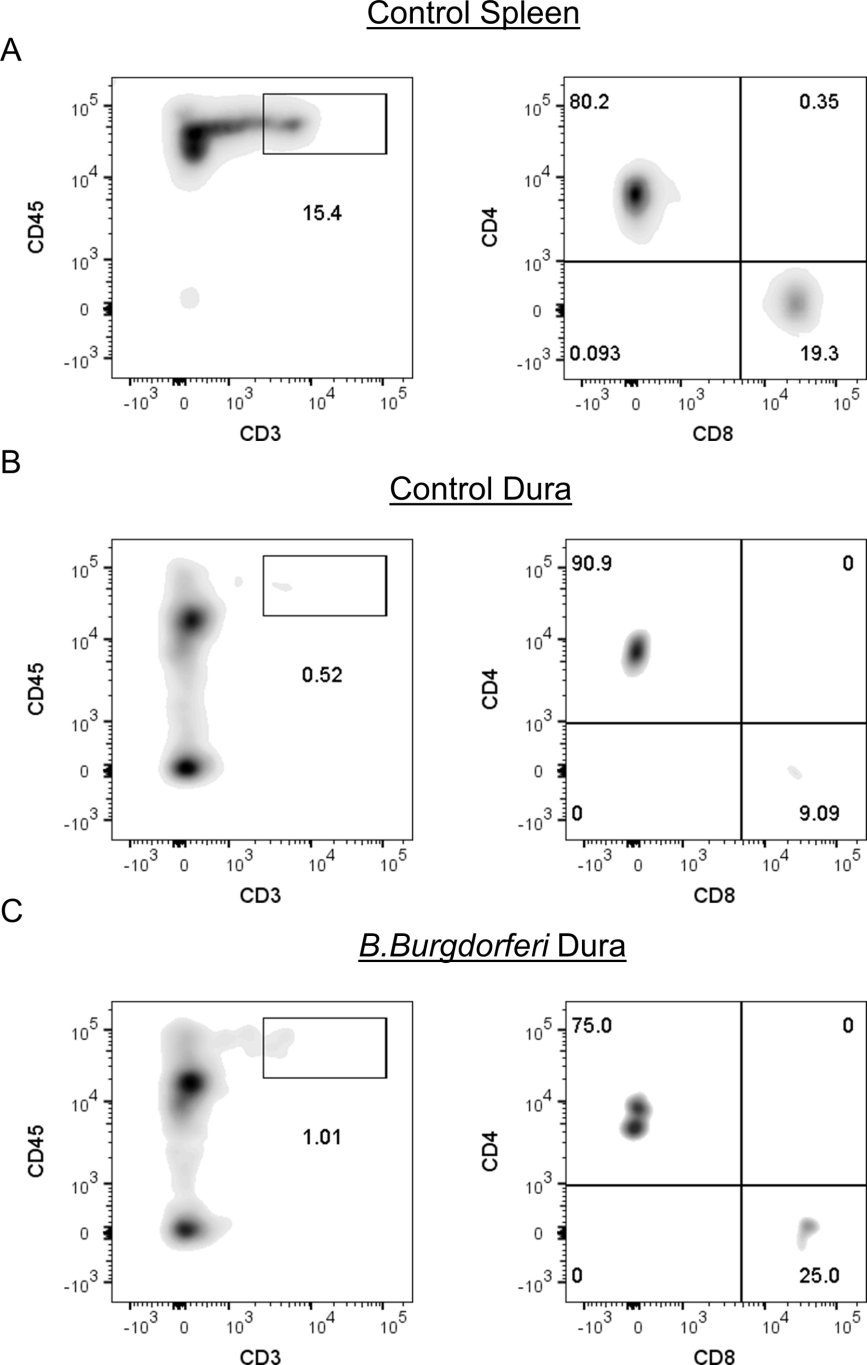
T cells in dura mater are CD4 and CD8 T cells. (A-C) Representative flow cytometric density plots showing gating strategy for the identification of CD4+ and CD8+ T cell subsets in the spleen (A) and dura mater (B-C). (A) CD3+ T cells (left), and CD4+ / CD8+ subsets (right) in splenocytes of control mice. (B/C) CD3+ T cells and CD4+/CD8+ subsets in dura mater of (B) control mice, and (C) *B*. *burgdorferi* infected mice. Prior to analysis all samples were gated on singlets using FSC-A vs FSC-H, and live cells were identified using Aqua amine reactive dye.

Immune responses to *B*. *burgdorferi* play a critical role in controlling infection, yet they are insufficient for complete resolution of borreliosis [27–29]. Because our infected animals had relatively low numbers of spirochetes in the dura mater at 75 dpi, we expected that there would be differences in lymphocyte numbers within the dura mater, suggestive of adaptive immune responses to infection. Given the importance of T cells in in adaptive immune responses to *B*. *burgdorferi* [30–36] we performed IHC on CNS tissues from infected mice at 75 dpi, and age matched controls (n = 3 per group). Spleens from infected mice were used as positive controls for CD3 staining, and isotype controls on brain samples showed no evidence of CD3+ cells (S3A–S3C Fig). Consistent with our expectations, we observed increased numbers of T cells in the brain vasculature (p = 0.0972), a statistically significant increase in T cells in the pia mater (p = 0.0050), and a statistically significant increase in T cells in the dura mater (p = 0.0403) (Fig 3A–3D, 3F and 3G). T cells were not observed in the brain parenchyma, and although T cells were detected in extravascular regions of the dura mater (Fig 3E), the number of T cells in the extravascular regions was not significantly different from control samples (p = .3739). To determine the identity of the CD3+ T cells in the dura mater, we performed flow cytometry on single cell suspensions of dura mater. Our results demonstrated that the CD3+ T cells in the dura of control and infected animals consisted of both CD4 and CD8 T cells (Figs 4A–4C and S4A and S4B). The differences in the frequency of CD8 and CD4 T cells between infected and control samples were not statistically significant (S4C and S4D Fig). Overall, the increase in the number of T cells in the meninges is suggestive of adaptive immune responses to *B*. *burgdorferi* colonization of the dura mater.

## Discussion

We initially expected that strain 297 would colonize the dura mater, because it is a clinical CSF isolate that is thought to be neurotropic [20]. In contrast, strain B31 is an isolate that originally came from a tick, and has not previously been shown to have tropisms for CNS tissues. The observation that both B31 and 297 colonized the dura mater suggests that these strains may not have differences in tropisms for CNS tissues, and that the dura mater is potentially a general site of colonization for *B*. *burgdorferi sensu stricto* in a murine system.

Multiple studies have demonstrated that the ability to enter blood circulation, followed by adhesion to blood vessels, is a key determinant in the survival and dissemination of *B*. *burgdorferi*, and other bacterial pathogens [37–39]. In the absence of vascular adhesion molecules, especially fibronectin binding proteins, *B*. *burgdorferi* demonstrates delayed dissemination to peripheral tissues [40–42]. It has been shown that *B*. *burgdorferi* must adhere to the vasculature with sufficient affinity and for a long enough duration to extravasate through the endothelial junctions of distal tissues while withstanding the shear forces of blood flow [42]. Not surprisingly, *B*. *burgdorferi* has been shown to adhere to post-capillary venules, as these areas have relatively lower shear forces than arterial blood [43, 44]. Consistent with the literature, the diameters of the vessels in the dura mater in which we observed borrelia adhering to were in the range of 10-30um, and had morphology that was consistent with capillaries, or post-capillary venules. Although it is common to see spirochetes adhering to blood vessels during acute infection, we expected that by 75 dpi, the majority of spirochetes would be found in the extracellular matrix, as these areas are less exposed to blood, and are thought to be a better protective niche for evading humoral immune responses [45]. Contrary to our expectations, most spirochetes were found adhering to blood vessels in the dura mater, suggesting that at this time point, *B*. *burgdorferi* antibody avoidance mechanisms are sufficiently robust to render humoral immunity ineffective[46].

Few individuals have reported culture positivity from the brains of mice infected by *B*. *burgdorferi* [9–11]. Our qPCR data support our culture results that were obtained on 45 dpi in strain 297, but it seems that for strain B31, the culture positives were due an infection burden that was below our limit of detection by PCR. Because all of our samples had been perfused prior to culture, we expected that any culture positive results were likely due to spirochetes adhering to blood vessels in the brain, or associated with the pia mater. Our IHC approach on samples from 75 dpi was sufficient for detecting CD3+ T cells in brain sections, however we did not detect any spirochetes in brain sections of mice. Again, these results could be due either to lack of spirochetes in the brains at this time point, or too few spirochetes present to be detected by our methods. Overall it seems that the brain is a rare target of *B*. *burgdorferi* colonization, and the precise region(s) of brain colonization remain elusive.

While both B and T cells are important effectors in controlling immune responses to borreliosis, we focused on T cells, because they are known to traffic through the lymphatics in the dura mater, even under homeostatic conditions [14]. Consistent with the literature, we observed CD3+ cells in the dura mater in both control and infected tissues, many of which were associated with lymphatic-like vessels that run parallel to the vascular sinuses (Fig 3B). The T cells that we observed in the dura mater were either adhering to vessels, or present as single cells in the extravascular spaces. We did not observe any T cell clustering, or any evidence of ectopic lymphoid tissue formation, suggesting that the dura mater was not likely to be the primary tissue associated with initial T cell activation, as would be expected in a lymph node. The observation that more T cells were detected adhering to the dura mater at 75 dpi when *B*. *burgdorferi* colonization was evident, suggests that the T cells may have been responding to the infection in this tissue. The low number of spirochetes detected in the dura at this time point are further supportive that immune responses are controlling, but not eliminating bacterial colonization of the dura mater.

In larger animal models, as well as in the clinical setting, neuroinflammation is typically measured by changes in the number of leukocytes or cytokine levels present in the CSF. Although we did not sample CSF in the present study, our future studies will focus on quantifying differential changes in leukocytes and cytokine expression in CSF[47], and comparing those results changes in the meninges. The advantage of using mice in this approach is that transgenic and genetic knockout mutants could be used to investigate specific mechanisms of *B*. *burgdorferi* pathogenesis and associated neuropathologies. Antibiotic dosage and efficacy could be evaluated in the context of meningeal infection, as has been done in peripheral tissues [48].

We conclude that the dura mater provides a sufficient environment for *B*. *burgdorferi* colonization during disseminated borreliosis. Moreover, we show that T cells are increased in number in the infected dura, and in the pia mater, implying that at the time points we tested, they play a role the immune responses within the CNS. Collectively, our results suggest that there are aspects of *B*. *burgdorferi* meningeal infection that can be modelled in laboratory mice. We anticipate that this model will be useful for future investigations pertinent to mechanisms that underlie CNS immune responses that are associated with the control and clearance of *B*. *burgdorferi* in the dura mater.

## Supporting information

S1 Fig At the time point of 75dpi spirochetes (Bb) were not observed in association with the lymphatic-like vessels (LYVE-1) that run parallel to the sagittal sinus (SS, arrow) of the dura mater. Blood vessels were stained by antibodies to CD31.
(TIF)

S2 Fig (A) epifluorescence image of blood vessel in dura mater from unperfused mouse injected intravenously with 70 kilodalton dextran (red). (B) epifluorescence image of blood vessel in dura mater from mouse injected intravenously with 70 kilodalton dextran followed by perfusion. Nucleated cells are shown by DAPI staining (blue).
(TIF)

S3 Fig (A) CD3 positive control costained with DAPI showing T cell zone in the spleen of a *B*. *burgdorferi*-infected mouse. (B) Isotype control in spleen showing no background fluorescence in CD3 channel. (C) Isotype control in brain costained with DAPI and CD31, showing minimal background fluorescence in CD3 channel.
(TIF)

S4 Fig (A-B) flow cytometric gating strategy for the identification of singlets (A), and live cells (B). (C-D) Frequency of CD8 T cells (C), and CD4 T cells (D), detected by flow cytometry in the dura of control and *B*. *burgdorferi*-infected mice; n = 5, p = 0.9803, and 0.9376, respectively.
(TIF)
