# Supplementary figures and images for: Borrelia burgdorferi adhere to blood vessels in the dura mater and are associated with increased meningeal T cells during murine disseminated borreliosis
Source: PLoS One. 2018 May 3;13(5):e0196893. doi: 10.1371/journal.pone.0196893 (PMC5933741; doi:10.1371/journal.pone.0196893)

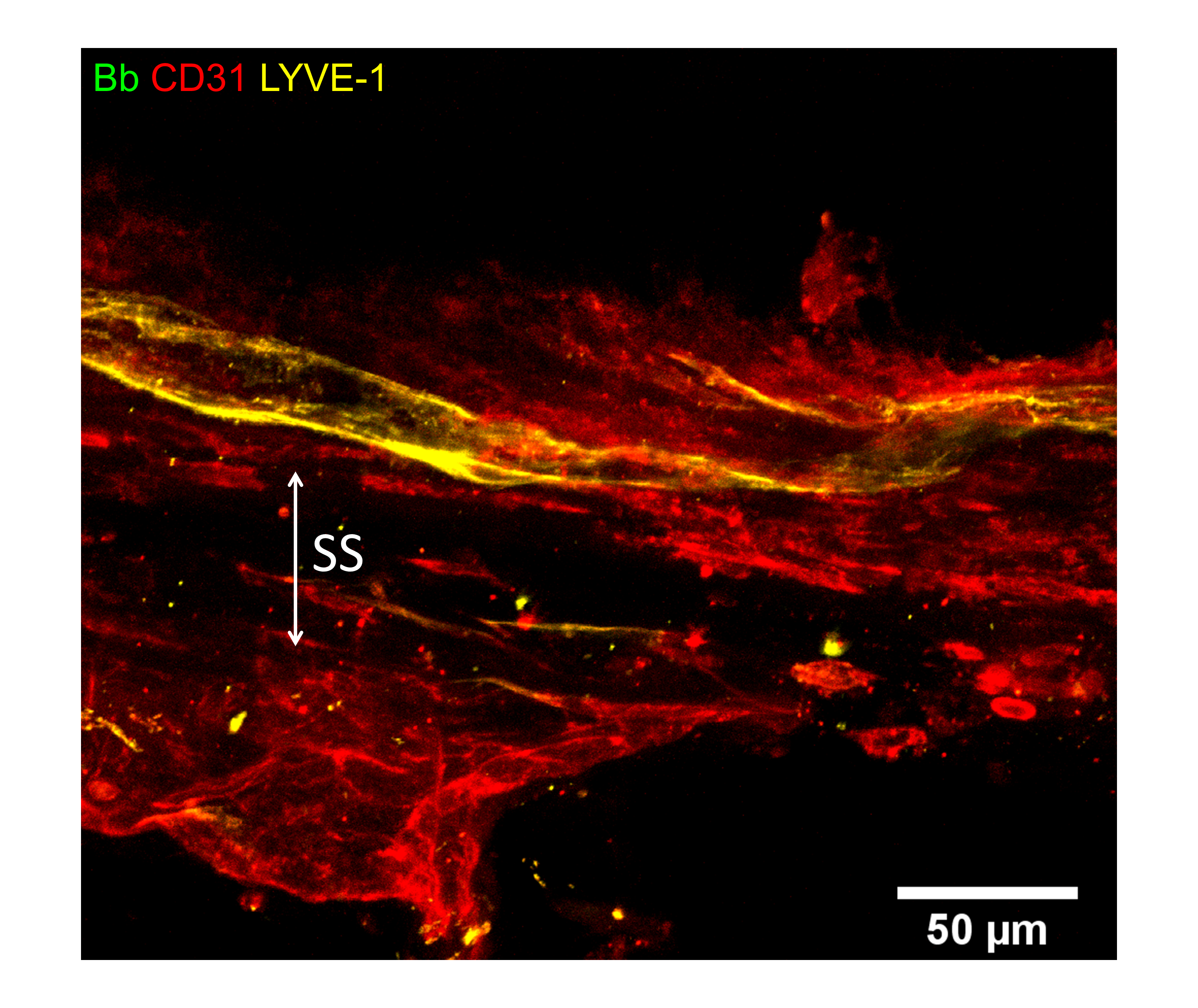

Supplement: S1 Fig — Blood vessels were stained by antibodies to CD31. (TIF) [file pone.0196893.s001.tif]

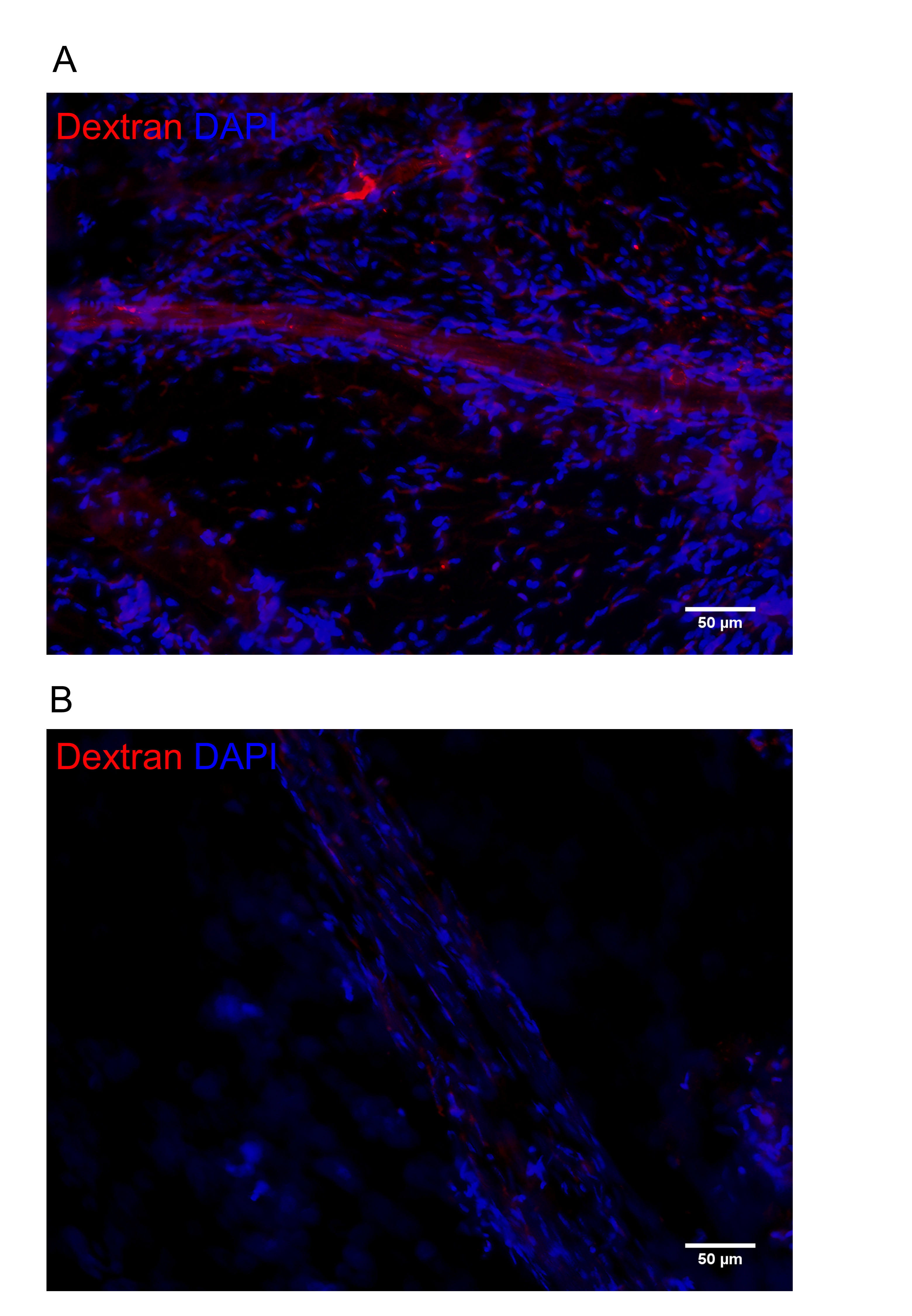

Supplement: S2 Fig — (A) epifluorescence image of blood vessel in dura mater from unperfused mouse injected intravenously with 70 kilodalton dextran (red). (B) epifluorescence image of blood vessel in dura mater from mouse injected intravenously with 70 kilodalton dextran followed by perfusion. Nucleated cells are shown by DAPI staining (blue). (TIF) [file pone.0196893.s002.tif]

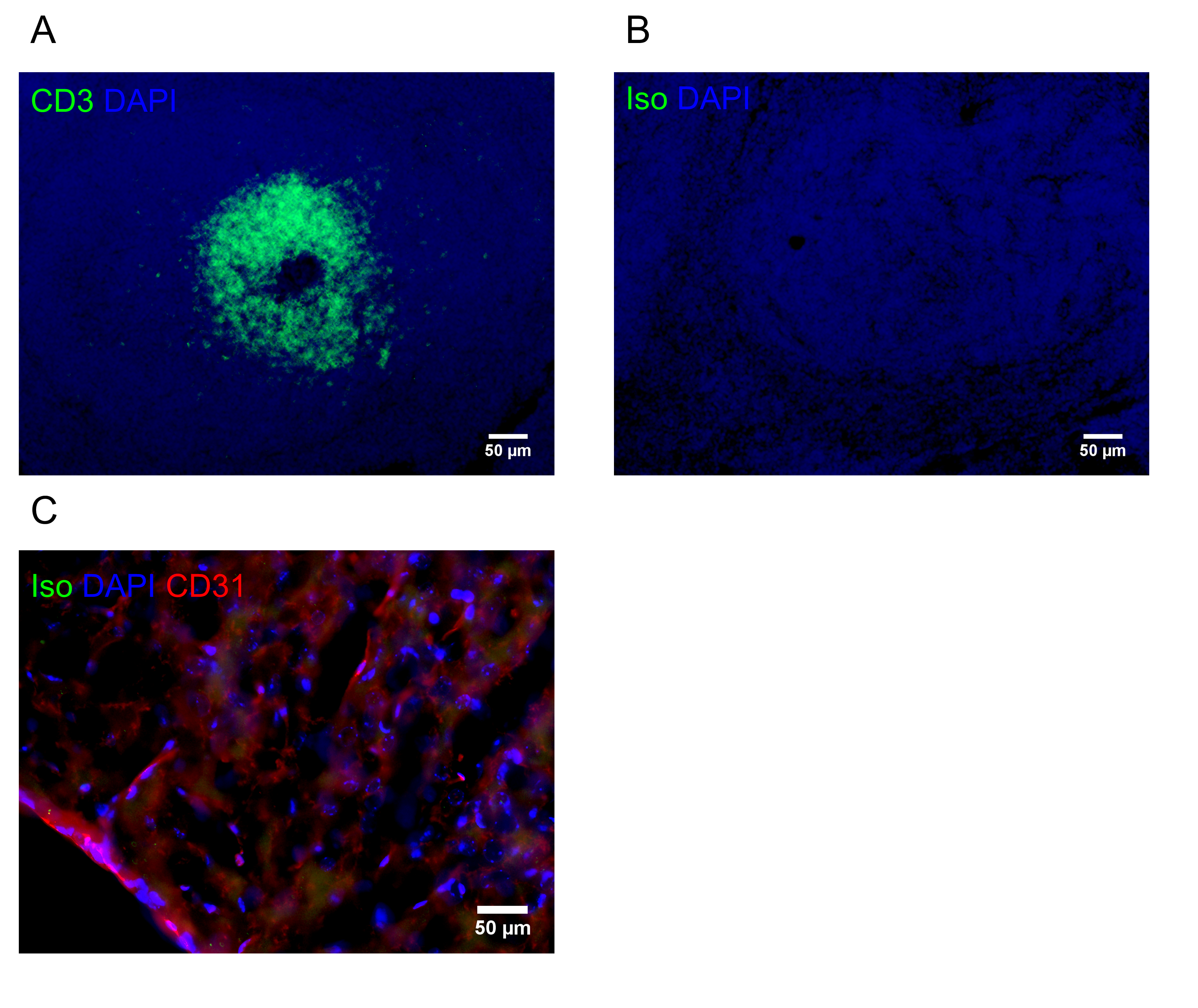

Supplement: S3 Fig — (A) CD3 positive control costained with DAPI showing T cell zone in the spleen of a B. burgdorferi-infected mouse. (B) Isotype control in spleen showing no background fluorescence in CD3 channel. (C) Isotype control in brain costained with DAPI and CD31, showing minimal background fluorescence in CD3 channel. (TIF) [file pone.0196893.s003.tif]

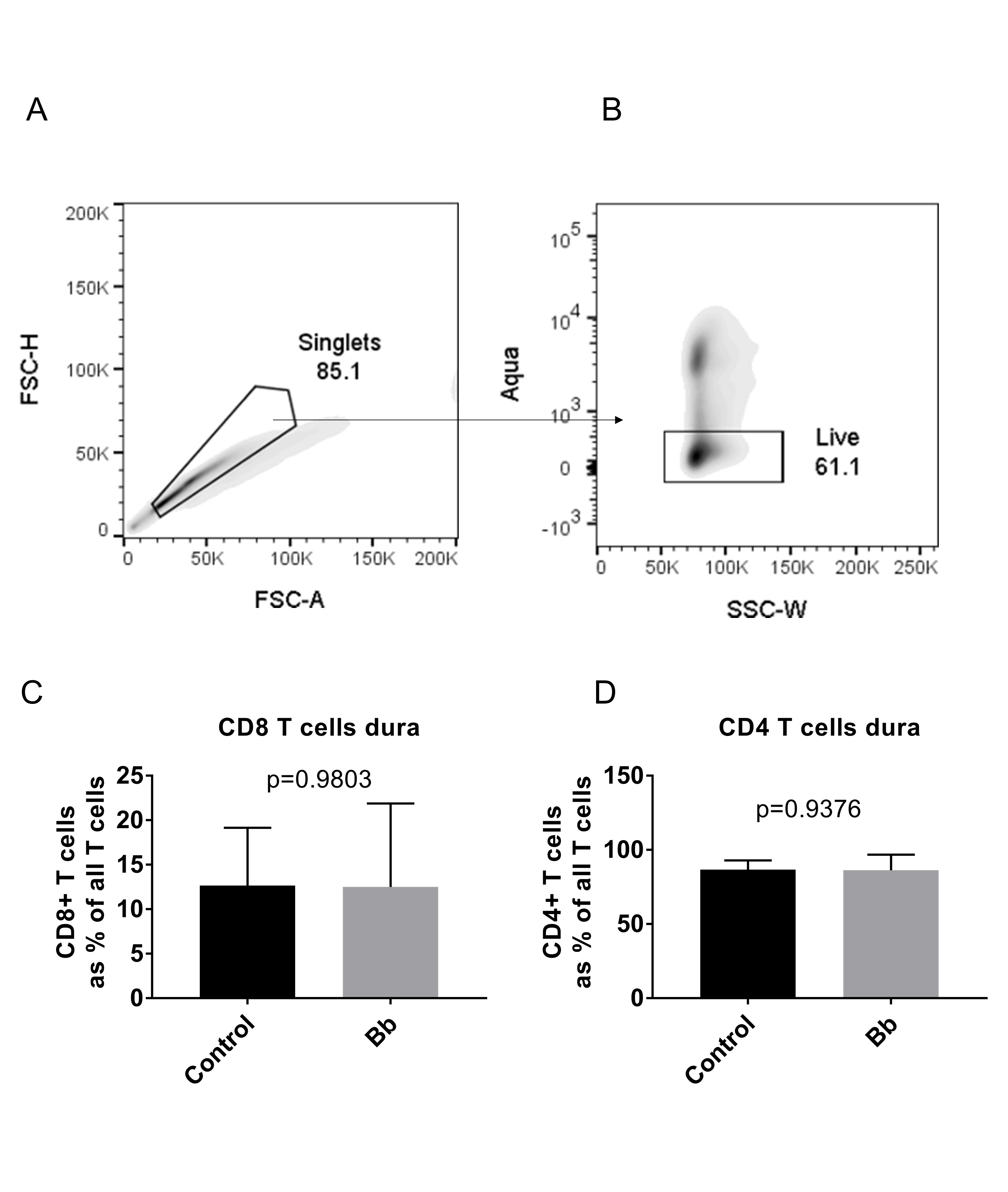

Supplement: S4 Fig — (A-B) flow cytometric gating strategy for the identification of singlets (A), and live cells (B). (C-D) Frequency of CD8 T cells (C), and CD4 T cells (D), detected by flow cytometry in the dura of control and B. burgdorferi-infected mice; n = 5, p = 0.9803, and 0.9376, respectively. (TIF) [file pone.0196893.s004.tif]
